# Supplementary material for: The genome sequence of the darkling beetle, Phaleria cadaverina (Fabricius, 1792) (Coleoptera: Tenebrionidae)
Source: Wellcome Open Res. 2026 May 11;11:269. [Version 1] doi: 10.12688/wellcomeopenres.26514.1 (PMC13320122; doi:10.12688/wellcomeopenres.26514.1)
Supplement: Peer review history

# Reviewer report: Reviewer response for version 1

**DOI:** 10.21956/wellcomeopenres.29199.r157898

**Published:** 2026-06-30

**Authors:** Ian M Carr

**Affiliations:** 1 University of Leeds, Leeds, UK

This report was well written and covered all the required information, and I have only two minor points. 

  The manuscript does not give the manufacturer of the PowerMasher, Qubit Fluorometer, femtoPulse or nanodrop instruments.

 Due to accessibility reasons, I have to copy and paste text to see it better and the PDF file misses out spaces in the text meaning it is harder to read. I believe this is due to the way the PDF was created; consequently, the PDF should be rebuilt.

Are sufficient details of methods and materials provided to allow replication by others?

Yes

Is the rationale for creating the dataset(s) clearly described?

Yes

Are the datasets clearly presented in a useable and accessible format?

Yes

Are the protocols appropriate and is the work technically sound?

Yes

Reviewer Expertise:

NGS data analysis, genome assembly, eDNA and medical genetics - Bioinformatics

I confirm that I have read this submission and believe that I have an appropriate level of expertise to confirm that it is of an acceptable scientific standard.

# Reviewer report: Reviewer response for version 1

**DOI:** 10.21956/wellcomeopenres.29199.r157889

**Published:** 2026-06-29

**Authors:** Gregor Bucher

**Affiliations:** 1 University of Göttingen, Göttingen, Germany

The authors present the genome sequence of the darkling beetle, *Phaleria cadaverine, *which was produced as part of the Darwin Tree of Life project. The quality of the chromosome level is very high and the required information is given (e.g. Description of the specimen, quality metrics, BUSCO analyses, etc.) and the data is made open access. The work represents a pure genome paper without further analyses.

 Two minor comments:

 - Full stops are missing before and after this phrase: “The order and orientation of scaffolds on Chromosome 10 between approximately 1–2.2 Mb is uncertain”

 - I guess that the sex of the individual used for Hi-C was a female as well?

Are sufficient details of methods and materials provided to allow replication by others?

Yes

Is the rationale for creating the dataset(s) clearly described?

Yes

Are the datasets clearly presented in a useable and accessible format?

Yes

Are the protocols appropriate and is the work technically sound?

Yes

Reviewer Expertise:

Gene function studies in beetles, evolution of head and brain development, RNAi in pest control

I confirm that I have read this submission and believe that I have an appropriate level of expertise to confirm that it is of an acceptable scientific standard.

# Reviewer report: Reviewer response for version 1

**DOI:** 10.21956/wellcomeopenres.29199.r155802

**Published:** 2026-06-03

**Authors:** Fahad Alqahtani

**Affiliations:** 1 King Abdulaziz City for Science and Technology, Riyadh, Saudi Arabia

The author of “The genome sequence of the darkling beetle, Phaleria cadaverina (Fabricius, 1792) (Coleoptera: Tenebrionidae)” successfully reconstructed the genome of the darkling beetle in chromosomal-level using PacBio HiFi and Hi-C sequencing. The assembly shows high quality with a length of 367.3 Mb and an impressive BUSCO completeness score of 99.7%

 However, I have several points that I believe should be addressed:

 1) The author states that an adult female specimen was used for genome sequencing and assembly. However, a second specimen was used for Hi-C sequencing, and the sex and life stage of this specimen are not reported. Please provide the sex and life stage for the Hi-C specimen.

 2)  It is recommended that the author clarify the reasoning behind the use of two different specimens for PacBio HiFi sequencing and Hi-C sequencing, rather than using the same specimen for both.

 3) Figure 1 shows only the specimen used for genome sequencing. Since a second specimen was used for Hi-C sequencing, the author should consider providing a photograph of the Hi-C specimen, if available.

 4) There appear to be minor differences between the chromosome lengths reported in the manuscript and those available in NCBI, specifically for chromosomes 3 and 5. Please verify and reconcile these lengths.

Are sufficient details of methods and materials provided to allow replication by others?

Yes

Is the rationale for creating the dataset(s) clearly described?

Yes

Are the datasets clearly presented in a useable and accessible format?

Yes

Are the protocols appropriate and is the work technically sound?

Yes

Reviewer Expertise:

Bioinformatics

I confirm that I have read this submission and believe that I have an appropriate level of expertise to confirm that it is of an acceptable scientific standard, however I have significant reservations, as outlined above.
